# Hidden Markov models for presence detection based on CO_2_ fluctuations

**DOI:** 10.3389/frobt.2023.1280745

**Published:** 2023-10-16

**Authors:** Christos Karasoulas, Christoforos Keroglou, Eleftheria Katsiri, Georgios Ch. Sirakoulis

**Affiliations:** Department of Electrical Computer Engineering, Democritus University of Thrace, Xanthi, Greece

**Keywords:** hidden Markov models, presence detection, carbon dioxide monitoring, motion sensors, Markov chain algorithms

## Abstract

Presence sensing systems are gaining importance and are utilized in various contexts such as smart homes, Ambient Assisted Living (AAL) and surveillance technology. Typically, these systems utilize motion sensors or cameras that have a limited field of view, leading to potential monitoring gaps within a room. However, humans release carbon dioxide (CO_2_) through respiration which spreads within an enclosed space. Consequently, an observable rise in CO_2_ concentration is noted when one or more individuals are present in a room. This study examines an approach to detect the presence or absence of individuals indoors by analyzing the ambient air’s CO_2_ concentration using simple Markov Chain Models. The proposed scheme achieved an accuracy of up to 97% in both experimental and real data demonstrating its efficacy in practical scenarios.

## 1 Introduction

The ability to locate individuals within their own residence has a multitude of applications, including the automation of tasks, securing doors when no one is home, detecting unauthorized presences, monitoring human activity, and identifying potential help situations, particularly for the elderly (Wilhelm et al., 2020). The conventional method for detecting individual’s presence in a room is through infrared motion sensors and image-processing systems such as surveillance cameras. However, these systems have limitations, as they can only detect active movement and are often expensive, computationally intensive and have a restricted field of view, resulting in monitoring gaps within a room. On the other hand, carbon dioxide (CO_2_) is a byproduct of respiration and is released into the ambient air. By monitoring the concentration of CO_2_, individuals’ presence or absence in a room can be determined with simple, low-cost CO_2_ sensors.

Multiple sensors can be used for detecting the human presence in indoor environments, including passive infrared (PIR) sensors (Dodier et al., 2006), video cameras (Yoshinaga et al., 2010; Di Lascio et al., 2013), infrared cameras, light beams placed in door frames and device-free localization that is based on radio signals (Vance et al., 2010).

The literature on occupancy patterns includes several studies that have used different approaches to simulate and estimate occupancy in single-office rooms and multi-tenant office buildings including those that have implemented this paper’s methods implementing Markov Chains. For instance, in (Sandels et al., 2015), the transition probabilities of the Markov chain were calculated using data obtained from occupancy sensors installed in 24 single office rooms within a Swedish building. To assess the model’s performance, the simulation results were compared to actual occupancy data from 23 other rooms in the same building. The analysis revealed that the model successfully replicated crucial features of the observed occupancy patterns. One study (Page et al., 2008) presents a non-homogeneous Markov chain model that simulates the times of arrivals and departures and the duration of intermediate absence and presence for individuals in single office rooms. Another study (Yu, 2010) uses a genetic programming algorithm to estimate similar behaviors with comparable accuracy to the previous study (around 80%–83%). A statistical approach to simulate the occupancy in single office rooms is presented in yet another study (Wang et al., 2005) where the authors show that the time duration of vacancies follows an exponential distribution, though this cannot be verified for time durations of occupancies. Another approach, a stochastic agent-based simulation model, is introduced in (Liao and Barooah, 2010) which simulates individual occupancy patterns between different zones of an office building with an error rate of 1% achieved in validation for one office room. A study (Duarte et al., 2013) applies a data mining approach to derive aggregated occupancy diversity factors from sensor data located in different room types of a large multi-tenant office building. In a research study (Wang et al., 2018), the use of WiFi probe technology was explored to collect and analyze connection requests and responses in order to monitor and evaluate the occupancy information of a building in real-time using feedback recurrent neural network (M-FRNN).

CO_2_ concentration-based methods have been frequently employed as a popular approach for estimating occupancy in various studies (Wang and Jin, 1998; Wang et al., 1999; Ansanay-Alex, 2013). However, these methods have certain drawbacks such as low sensitivity to larger areas, prediction latency and high initial installation costs without requiring additional infrastructure (Wang and Shao, 2016; Wang et al., 2017). Another widely applied indirect occupancy estimation approach uses carbon dioxide (CO_2_) concentration in indoor spaces (Wang and Jin, 1998; Wang et al., 1999; Jiang et al., 2016). For example, Wang et al. developed several dynamic CO_2_-based models for commercial buildings (Wang and Jin, 1998; Shan et al., 2012). Diaz and Jimenez conducted an experiment on the power usage of computers under occupancy variation estimated by CO_2_ and the results suggested that CO_2_ concentration is informative and expected to be a good indicator of occupancy [11].

Although increasing number of modern buildings utilize CO_2_ as reference in system control, CO_2_-based approaches have constraints, such as low sensitivity to occupant mobility and slow response to drastic occupancy changes (Yang et al., 2016). Wang et al. (Yang and Becerik-Gerber, 2014) found a suitable algorithm with the exhaust CO_2_ level as input. After implementing three versions of the direct approach based on the CO_2_ level, the result showed high accuracy. Similar studies were also presented in study of Mumma (Yang et al., 2016), the CO_2_ levels were measured in the room in the exhaust air, and the result showed fast estimations with accuracies of ±2 people. Carbon dioxide (CO_2_) sensors have been employed to estimate the number of occupants in the spaces for a long time (Shan et al., 2012; Ekwevugbe et al., 2013). As discussed earlier, occupancy is the CO_2_ generator and the occupancy presence can be inferred through the CO_2_ concentrate level. However, limitations such as the window or door positions, outdoor air supply rate, and the proximity of the occupants to the sensor have been reported. Uncertainties in the estimation errors, and second, latency of the CO_2_ sensor responses (i.e., the aspect of time delay) are also part of the limitation when applying CO_2_ sensor for occupancy number calculation (Ekwevugbe et al., 2013). CO_2_ sensors provide concentration readings in parts per million (ppm), which is indicative of the occupancy. However, reliably correlating CO_2_ levels with the actual occupancy is difficult due to the high variability and slow response time of CO_2_ sensors. Variability arises due to fluctuations in ambient CO_2_ levels, HVAC system settings, and door status (open/close). In addition, there are dynamics: CO_2_ measurements suffer from slow response time. For example, the inevitable delay in CO_2_ concentration increase following an increase in occupancy.

Other environmental sensors, such as temperature, humidity, lighting, and acoustic sensors, also can help improve occupancy prediction accuracy (Ekwevugbe et al., 2013; Yang and Becerik-Gerber, 2014). Based on these sensory data sources, researches proposed quantitative models to infer the number of occupants in a given space. Yang and Becerik-Gerber formulated stochastic processes that combining regression, time-series modeling, and pattern recognition modeling approaches to improve accuracy in occupancy prediction from a data analytic perspective (Yang and Becerik-Gerber, 2014). Jiang et al. proposed a feature scaled extreme learning machine (FS-ELM) approach on CO_2_ concentration to predict occupancy (Jiang et al., 2016).

The use of carbon dioxide (CO_2_) concentration as an indirect occupancy estimation approach in indoor spaces is widely applied. CO_2_ sensors provide absolute concentration readings in parts per million (ppm), which is indicative of the occupancy. CO_2_ sensors operate passively and do not require direct interaction with individuals in the room. This non-intrusive nature ensures that occupants are not disturbed or influenced by the sensing process making it particularly suitable for environments where privacy or comfort is a concern, something that vision sensors struggle with. Vision sensors may encounter challenges in scenarios with visual obstructions, such as curtains, furniture or may be affected by variations in lighting conditions. CO_2_ sensors are not hindered by physical barriers. Vision sensors may encounter challenges in scenarios with visual obstructions, such as curtains, furniture, or partitions. CO_2_ sensors, on the other hand, are not hindered by physical barriers and thus can effectively gauge occupancy levels regardless of visual obstructions.

The proposed setup holds significant potential for effective deployment in manufacturing environments, particularly in the context of Industry 4.0 advancements. CO_2_ sensors offers a promising alternative to certain vision-based applications commonly employed in industrial settings that can encounter challenges related to visual obstructions, lighting conditions, and privacy concerns. Their proficiency lies in their ability to gauge occupancy levels based on the fundamental physiological process of human respiration, rendering them unaffected by visual barriers or lighting variations.

However, reliably correlating CO_2_ levels with the actual occupancy is difficult due to the high variability and second, high latency of the CO_2_ sensor responses while uncertainties in the estimation errors have been reported in several cases. Some works address the above issues by fusing CO_2_ sensors with RF tags, IR sensors and more recently WiFi terminals and GPS. However although this approach generally increases the estimation accuracy, it is not practical as not only it increases also the installation cost but it is invasive on the user, who often needs to carry around additional devices. This work addresses the above issues by implementing a sensor-driven occupancy estimation approach based on a) data point-level differences in CO_2_ concentrations, b) a reliable real-time CO_2_ sensing device that uses low-cost sensor technology, c) a simple HMM modelling approach.

This research paper focuses on the use of a CO_2_ sensor as a cost-effective and reliable method for detecting the presence or absence of individuals in a room. The proposed approach is based on a simple Markov Chain model which can be easily and inexpensively implemented and adapted to any environment. Unlike previous studies, the present research demonstrates that the CO_2_-based approach can achieve higher accuracy in detecting occupancy without the need for additional PIR, image, or environmental sensors (Wang et al., 2018). Moreover, the proposed method was tested and validated using both real-world and experimental data, demonstrating its potential for practical applications. The primary motivation for this study was to explore the feasibility and effectiveness of using a single CO_2_ sensor to detect occupancy in situations where other sensing technologies are not available. The results of this study offer promising insights into the use of CO_2_-based sensing for occupancy detection in various indoor environments. Given the sensor’s remarkable high-response capabilities it presents significant potential for real-time applications. In particular, by employing the following algorithms, it becomes feasible to trigger specific indoor functions such as HVAC and lighting bulbs. Consequently, the sensor can be powered down after each use, effectively extending its operational lifespan. This approach not only maximizes the sensor’s utility but also optimizes its longevity.

The remaining of the paper is structured as follows: Data collection methods are presented in Section 2. Section 3 presents Hidden Markov modeling methodology, describing the algorithms and the classification rules utilized for occupancy detection in both experimental and real data. Section 4 presents the results that both algorithms produce. Comparison of the algorithm’s parameters are discussed in Section 5 and the paper concludes with Section 6.

## 2 Materials and methods

### 2.1 CO_2_ measurements

The research was conducted in two stages: first, occupancy detection of an office room was achieved by analyzing a dataset that contained light, temperature, humidity, and CO_2_ measurements using statistical learning models from a GitHub dataset (https://github.com/LuisM78/Occupancy-detection-data) that was successfully implemented in the findings of (Candanedo and Feldheim, 2016) and only CO_2_ measurements were extracted for this paper’s purpose. Subsequently, real-world data was collected from a public service building in Athens, Greece, that was occupied during weekday mornings and remained unoccupied on weekends.

A measuring device Siba C2O2O: CO, CO_2_ that was developed in the auspices of the “Air-19” project for monitoring both air quality and public health at the Decentralised Administration of Athens, was employed to record the data. This method exploits low-cost sensor technology and integrates pre-calibrated sensors that provide certified measurement.The range of the CO_2_ sensor is 0–5000 ppm and it has a response time < 30 s (from 0 to 10 ppm) and very low noise (ppb equivalent). The C2O2O device was perfectly pre-calibrated by its manufacturer and was mounted on a vertical wall on the 3rd floor of the building which hosts an area with two cashier booths, where visitors queue to pay their council tax. The tills are at the center of an open-space office area with 20 desks. The device is configured to connect to a WiFi network, dedicated for IoT traffic to communicate the measurements to a cloud platform for storage and visualisation. The measurements selected for this pilot were collected from November 1st to 31 November 2022. The area opens at 6:00 a.m. by the cleaners, receives visitors between 7a, and 3p.m. when it closes for the public while around 4p.m. employees leave for the day. More information about this experiment can be found in (Katsiri, 2023).

The Markov Chain model used in this paper is simple and intuitive. The states directly correspond to meaningful conditions (higher, lower, equal CO_2_ levels) which makes the algorithms easily understandable. Markov Chains are also well-suited for modeling sequences of events which is exactly what the CO_2_ sensor data of this paper represents. The transitions between states capture the temporal aspect of the data that may be harder to model with a neural network which would also be overkill and more computationally demanding for only 400 measurements per testing set”.

### 2.2 Methodology

This research did not rely on CO_2_ concentrations to infer occupancy status. Instead, the focus was on detecting CO_2_ fluctuations and their relationship with occupancy. We simplify the problem expressing it as a Hypothesis testing problem. Specifically, we have 1) *H* ^1^: “at least one person is always present in the room during the whole duration of the measurements,” and 2) *H* ^2^: “no person is present at any time during the whole duration of the measurements” (*H* ^2^ complements *H* ^1^). To test *H* ^1^, and *H* ^2^ hypotheses we construct two Hidden Markov Models (*S* ^1^, and *S* ^2^) to capture CO_2_ behaviour regarding human presence (*S* ^1^), or absence (*S* ^2^). The HMMs consist of three states—“Equal,” “Plus,” and “Minus”—to analyze CO_2_ fluctuations over time. Specifically:

• Equal state represents the case when the absolute difference between the current CO_2_ value (i.e., *v*_*c*_) and its previous value (i.e., *v*_*p*_) is within a certain threshold^1^ (i.e., |*v*_*c*_ − *v*_*p*_| < *dif*).
• Plus state represents the case when the current CO_2_ value is above a certain range and higher than the previous value (i.e., *v*_*c*_ − *v*_*p*_ > *dif*).
• Minus state represents the case when the previous CO_2_ value is above the range and higher than the current value (i.e., *v*_*p*_ − *v*_*c*_ > *dif*).

#### 2.2.1 Hidden Markov models description

Definition 1 (*HMM Model*)*. An HMM is described by a five-tuple* *S* = (*Q*, *E*, Δ, Λ, *π*_0_)*, where* *Q* = {*q*_1_, *q*_2_, …, *q*_|*Q*|_} *is the finite set of states;* *E* = {*e*_1_, *e*_2_, …, *e*_|*E*|_} *is the finite set of outputs;* Δ: *Q* × *Q* → [0 1] *captures the state transition probabilities;* Λ: *Q* × *E* × *Q* → [0 1] *captures the output probabilities associated with transitions; and* *π*_0_ *is the initial state probability distribution vector. Specifically, for* *q* *,* *q*′ ∈ *Q* *and* *σ* ∈ *E* *, the output probabilities associated with transitions are given by*

$$\Lambda \left(q,\sigma ,q^{\prime }\right)\equiv \mathrm{Pr}\left(q\left[t+1\right]=q^{\prime },E\left[t+1\right]=\sigma |q\left[t\right]=q\right)\;,$$ (1)

and the state transition probabilities are given by

$$\Delta \left(q,q^{\prime }\right)\equiv \mathrm{Pr}\left(q\left[t+1\right]=q^{\prime }|q\left[t\right]=q\right)\;,$$ (2)

where *q*[*t*] (*E*[*t*]) is the state (output) of the HMM at time step (or epoch) *t*. The output function Λ(*q*, *σ*, *q*′) describes the conditional probability of observing the output *σ* associated with the transition to state *q*′ from state *q*. The state transition function needs to satisfy

$$\begin{array}{c} \Delta \left(q,q^{\prime }\right)=\underset{\sigma \in E}{\sum }\Lambda \left(q,\sigma ,q^{\prime }\right),\;\;\forall q,q^{\prime }\in Q \end{array}$$ (3)

and also

$$\overset{\left|Q\right|}{\underset{i=1}{\sum }}\Delta \left(q,q_{i}\right)=1,\;\;\forall q\in Q.$$ (4)

We also define $A_{e_{i}}^{\left(j\right)}$ the transition matrix for *S* ^(*j*)^, *j* = {1, 2}, under the output symbol *e*_*i*_ ∈ *E*. The matrix $A_{e_{i}}^{\left(j\right)}$ , is associated with output *e*_*i*_ ∈ *E* ^(*j*)^, as follows: the (*k*,*l*)^*th*^ entry of $A_{e_{i}}^{\left(j\right)}$ captures the probability of a transition from state *q*_*l*_ to state *q*_*k*_ that produces output *e*_*i*_, i.e., $A_{e_{i}}^{\left(j\right)}\left(k,l\right)=\Lambda^{\left(j\right)}\left(q_{l},e_{i},q_{k}\right)$ . We set $A_{e_{i}}^{\left(j\right)}\text{ to zero if }e_{i}\in E\backslash E^{\left(j\right)}$ .

We can calculate $P_{i}^{\left(j\right)}=\mathrm{Pr}\left(\omega \left(i\right)|S^{\left(j\right)}\right)$ with an iterative algorithm, a detailed description of which can be found in Athanasopoulou and Hadjicostis (2008); Fu (1982). Specifically, given sequence *ω* = *ω*[1]*ω*[2], …, *ω*[*n*] we calculate

$$\rho_{n}^{\left(j\right)}=A_{\omega \left[n\right]}^{\left(j\right)}A_{\omega \left[n-1\right]}^{\left(j\right)}\ldots A_{\omega \left[1\right]}^{\left(j\right)}\pi_{0}^{\left(j\right)},$$

which is essentially a vector whose *k* ^*th*^ entry captures the probability of reaching state *q*_*k*_ ∈ *Q* ^(*j*)^ while generating the sequence of outputs *ω* (i.e., $\rho_{n}^{\left(j\right)}\left(k\right)=\mathrm{Pr}\left(q\left[n\right]=q_{k},\omega |S^{\left(j\right)}\right)$ ). If we sum up the entries of $\rho_{n}^{\left(j\right)}$ we obtain $P_{\omega }^{\left(j\right)}=\mathrm{Pr}\left(\omega |S^{\left(j\right)}\right)=\sum_{k=1}^{\left|Q^{\left(j\right)}\right|}\rho_{n}^{\left(j\right)}\left(k\right)$ .

In the following example, we construct *S* ^1^, and *S* ^2^ hidden Markov models using a method that is commonly used in simple Markov chains (i.e., we estimate the transition probabilities counting the frequency of occurrences of each transition), due to the fact that in our example, the models are designed to capture observable states and their transitions without involving hidden or unobservable states. This approach allows us to focus on the direct dependencies and transitions between the states themselves, making the modeling process more straightforward and easier to interpret.

The simplicity of the method lends itself well to scenarios where the underlying system can be adequately represented using only observable information, and where the goal is to analyze and predict patterns based on the observed data. However, for more complex real-life scenarios, where unobservable transitions can occur, a more elaborate learning process should be used such as Baum-Welch algorithm Rabiner and Juang (1986). In our case, we have simple Markov chains with only 3 states. However, in a more general case, we could possibly need to answer to the question of how many states our model should have in order to decide correctly the human absence/or presence. This is in general a difficult problem, which is described in detail in Rabiner and Juang (1986).

**FIGURE 1:**
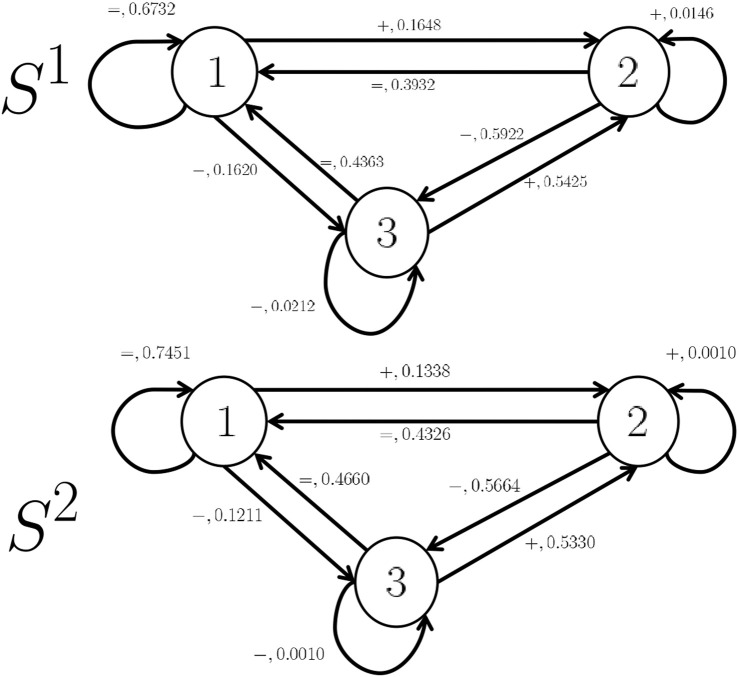
Occupied and Unoccupied Markov Chain models.

Example 1 *We construct two HMMs* (*S* ^1^ *, and* *S* ^2^) *as drawn in* Figure 1 *using appropriate training datasets from real-life scenarios (see* Section 4 *), where* $S^{1}=\left(Q,E,\Delta^{1},\Lambda^{1},\pi_{0}^{1}={\left[1\;0\;0\right]}^{T}\right)$ *, and* $S^{2}=\left(Q,E,\Delta^{2},\Lambda^{2},\pi_{0}^{2}={\left[1\;0\;0\right]}^{T}\right)$ *with*

• *Q* = {1, 2, 3}*, where state 1 represents the “Equal” state, state 2 represents the “Plus” state, and state 3 represents the “Minus” state,*
• *E* = {*“* =*”*, *“* +*”*, *“* −*”*}*, where event* *“* =*”* *means that the state 1 will be the next state, event* *“* +*”* *means that stste 2 will be the next state, and event* *“* −*”* *means that state 3 will be the next state* ^2^.
• *We define below all matrices* $A_{e}^{j}$ *for* *j* ∈ {1, 2}*, and* *e* ∈ *E* *:*

$$\;\begin{array}{ll} A_{“=”}^{\left(1\right)} & =\left[\begin{array}{ccc} 0.6732 & 0.3932 & 0.4363\\ 0 & 0 & 0\\ 0 & 0 & 0 \end{array}\right]\text{, }\\ A_{“+”}^{\left(1\right)} & =\left[\begin{array}{ccc} 0 & 0 & 0\\ 0.1648 & 0.0146 & 0.5425\\ 0 & 0 & 0 \end{array}\right] \end{array}$$

$$\;\begin{array}{ll} \;A_{“-”}^{\left(1\right)} & =\left[\begin{array}{ccc} 0 & 0 & 0\\ 0 & 0 & 0\\ 0.162 & 0.5922 & 0.0212 \end{array}\right],\\ A_{“=”}^{\left(2\right)} & =\left[\begin{array}{ccc} 0.7451 & 0.4326 & 0.466\\ 0 & 0 & 0\\ 0 & 0 & 0 \end{array}\right] \end{array}$$

$$\;\begin{array}{ll} \;A_{“+”}^{\left(2\right)} & =\left[\begin{array}{ccc} 0 & 0 & 0\\ 0.1338 & 0.001 & 0.533\\ 0 & 0 & 0 \end{array}\right]\text{, }\\ A_{“-”}^{\left(2\right)} & =\left[\begin{array}{ccc} 0 & 0 & 0\\ 0 & 0 & 0\\ 0.1211 & 0.5664 & 0.001 \end{array}\right]. \end{array}$$

Our analysis aims to classify between the two HMMs to identify which of the two Hypotheses is correct (*H* ^1^ or *H* ^2^). For that reason we use two classification methods developed in Section 3.2.

#### 2.2.2 Classification rules

Definition 2 *Optimal Decision Rule (MAP Rule). If we observe a sequence of* *n* *outputs* *ω* = *ω*[1]*ω*[2], …, *ω*[*n*]*, with* *ω*[*t*] ∈ *E* *, that is generated by one of the two underlying HMMs, we decide in favor of* *S* ^*j*^ *, where* *j* ∈ {1, 2} *if* $P_{j}P_{\omega }^{j}=\max\left\{P_{1}P_{\omega }^{1},P_{2}P_{\omega }^{2}\right\}$ *. Moreover, the probability of error in a classical hypothesis testing problem (Neyman, 1992) is given as* $P_{\mathrm{error}}\left(\omega \right)=\min\left\{P_{1}P_{\omega }^{\left(1\right)},P_{2}P_{\omega }^{\left(2\right)}\right\}$ . *We subsequently normalize the probability of error for any sequence introducing* *α*(*ω*) *which is given below* ^3^ *:*

$$\alpha \left(\omega \right)=\min\left\{\frac{P_{1}P_{\omega }^{\left(1\right)}}{P_{1}P_{\omega }^{\left(1\right)}+P_{2}P_{\omega }^{\left(2\right)}},\frac{P_{2}P_{\omega }^{\left(2\right)}}{P_{1}P_{\omega }^{\left(1\right)}+P_{2}P_{\omega }^{\left(2\right)}}\right\}\;.$$ (5)

We prefer to use *α*(*ω*) because it provides information on how good is our decision in terms of probabilistic ambiguity (i.e., if *α*(*ω*) is close to zero then we know that our decision is good. On the other hand, if *α*(*ω*) is close to 0.5 it means that we have large ambiguity in our decision).

Reducing the computational complexity of the optimal decision rule, we revisit an empirical sub-optimal decision rule which is described in Keroglou and Hadjicostis (2018). Roughly, the sub-optimal rule allows us to decide correctly the appropriate HMM only by counting the number of times that one/or more state(s) is/are visited.

Formally the suboptimal rule is described below. We define two metrics that are required for the definition of the suboptimal rule. Specifically, i) Fraction of times a state appears, and ii) distance in variation between two probability vectors.

• (Fraction of times state *i* appears (*m*_*n*_(*i*))). As an example, we define first *m*_*n*_(1). Suppose we are given an observation sequence of length *n* $\left(\omega_{1}^{n}=\omega \left[1\right]\cdots \omega \left[n\right]\right)$ . We define $m_{n}\left(1\right)=\frac{1}{n}\overset{n}{\underset{t=1}{\sum }}g_{1}\left(\omega \left[t\right]\right)$ , where

$g_{1}\left(\omega \left[t\right]\right)=\left\{\begin{array}{cc} 1,\; & \text{if }\omega \left[t\right]\text{ is }“{=}^{\prime \prime },\\ 0,\; & \text{ otherwise}. \end{array}\right.$

In other words, *m*_*n*_(1) is the fraction of times state 1 appears in observation sequence $\omega_{1}^{n}$ . Similarly, we define *m*_*n*_(2), and *m*_*n*_(3), for states 2, and 3 respectively.

• (Distance in variation *d*_*V*_(*v*, *v*′) between two probability vectors *v*, *v*′). The distance in variation (Dembo and Zeitouni, 1998) between two |*Q*|-dimensional probability vectors *v*, *v*′ is defined as

$$d_{V}\left(v,v^{\prime }\right)=\frac{1}{2}\overset{\left|Q\right|}{\underset{j=1}{\sum }}|v\left(j\right)-v^{\prime }\left(j\right)|\geq 0,$$ (6)

where *v*(*j*) (*v*′(*j*)) is the *j*th entry of vector *v* (*v*′).

Definition 3 (*Sub-optimal Empirical rule*)*. Given two HMMs* *S* ^(1)^ *and* *S* ^(2)^ *and a sequence of observations* $\omega_{1}^{n}=\omega \left[1\right]\omega \left[2\right]\cdots \omega \left[n\right]$ *, we perform classification using the following suboptimal rule:*

• *We first compute* $m_{n}={\left[m_{n}\left(1\right),m_{n}\left(2\right),m_{n}\left(3\right)\right]}^{T}$

• *We then set* $\theta =\frac{1}{2}d_{V}\left(\pi_{ss}^{\left(1\right)},\pi_{ss}^{\left(2\right)}\right)$ *, where* $\pi_{ss}^{\left(j\right)}$ *,* *j* ∈ {1, 2}*, is the steady-state probability vector for* *S* ^*j*^ *, and compare* $d_{V}{\left(m_{n},\pi_{ss}^{\left(1\right)}\right)}_{<}^{>}\theta$ *. We decide in favor of* *S* ^1^ (*S* ^2^) *if the right (left) quantity is larger* ^4^.

The empirical rule is a suboptimal rule, which means that even if we compute exactly the probability of error using the empirical rule, this remains an upper bound on the probability of error using the optimal rule. Using empirical rule has some advantages over the optimal rule. There are necessary and sufficient conditions for a bound on the probability of error using the empirical rule to be asymptotically tight (see Keroglou and Hadjicostis, 2018). Moreover, these conditions can be verified with low computational complexity (polynomial complexity). Another advantage is that the system needs to keep only the number of events that are observed and not the whole observation sequence. This can lead to lower memory requirements for the system.

In the suboptimal rule, we count the number of visits for all states (1, 2, and 3). However, for simplicity in the following results we apply the empirical rule only to one state. The most suitable state out of the three states for this purpose was identified as the state *j* ∈ {1, 2, 3} which maximizes the following quantity $\left|\pi_{ss,j}^{\left(1\right)}-\pi_{ss,j}^{\left(2\right)}\right|$ . The lower this difference is the sturdier the results are.

## 3 Results

The results section initiates with an empirical examination of data sourced from the meticulously calibrated sensors as detailed in the associated Github repository and the same methodology is applied to the CO_2_ sensor installed in Athens. The predominant focus revolves around the application of the Optimal Rule with the supplementary incorporation of the sub-optimal rule to augment the insights derived from the primary approach. The way and reasoning behind the splitting of the training and testing sets is analyzed and the upcoming results and benefits of having the identical time window training and testing sets are presented in the sub-sections below.

### 3.1 Optimal rule results

#### 3.1.1 Experimental data

Firstly, experiments are conducted using the experimental data and that is achieved through a straightforward procedure since the Github data points are less compared to the real sensor data taken in Athens and the time period between two data points is much higher. The main parameters that need to be taken into account and will result in different outcomes are the hours that will be utilized as training sets and the threshold that will determine if two consecutive measurements belong in the “equal” state (referred to as threshold “dif” [see Section 3)].

Some strict assumptions are necessary to be made in order for the experiment to make sense. When a day is referred to as “occupied” it means that there is at least one person present in the workspace at all times whereas “unoccupied” means there is nobody present. When CO_2_ measurements are analyzed only these 2 variables are taken into account and the number of occupants is unimportant for this research.

The best training hours of the GitHub dataset were identified as the work hours of 2015-02-16 for creating the occupied Markov Model and the work hours of 2015-02-15 for the unoccupied Markov Model. After using multiple thresholds (dif) for naming a state transition as “equal” some Markov Models yield better results than others.

**FIGURE 2:**
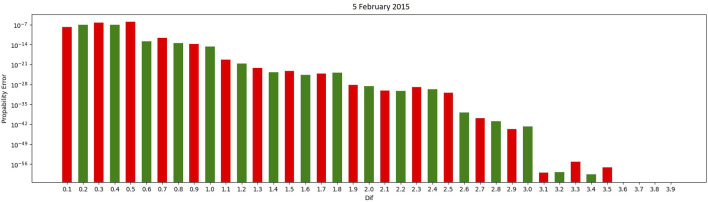
Dif graph using a threshold between two consecutive CO_2_ measurements.

In Figure 2, classifying a state transition as “equal” when two consecutive CO_2_ measurements have less than a 3.1 difference provided the best results for the specific testing set.

Markov Chain models using multiple thresholds categorizing an equal state had 100% success in accurately concluding the occupied state of all days in the testing sets provided.

#### 3.1.2 Real data

Real data was obtained by installing a CO_2_ sensor in a bank located in Athens, Greece. This sensor gathered data every 5 s throughout 1 month, from the 1st to the 30th of November 2022. During this time, the weekdays were deemed “occupied” and the weekends were considered “unoccupied”.

To create the occupied and unoccupied Markov Chain models, 2 days were selected as a training set, while the remaining days were allocated for testing purposes. A range of hours was implemented for the training sets, primarily consisting of 2-h windows that were tested against 1-h window testing sets, starting from 8 a.m. until 4 p.m. For example, the 10:00 to 12:00 timeframe on November 30th could be utilized as a training set and then tested against eight hourly testing sets on November 29th.

**FIGURE 3:**
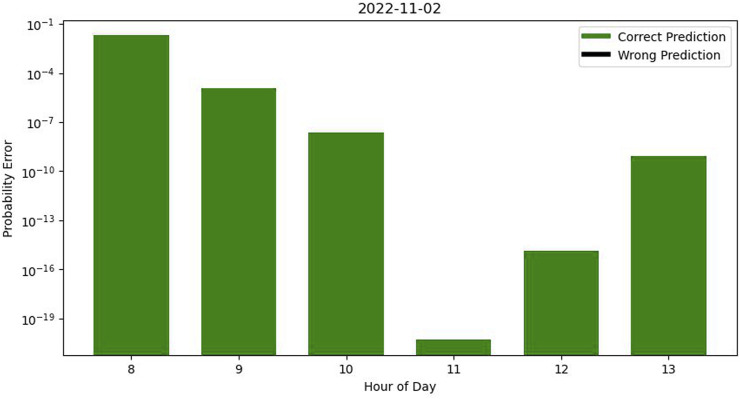
Error graph in a day with correct predictions.

If we aggregate the results on a daily basis by dividing each day into six testing sets spanning from 8 a.m. to 2 p.m., we can generate a graph to evaluate the accuracy and assess probability errors. For instance, on 2022-11-03, as illustrated in Figure 3, certain probability errors exhibit significantly smaller magnitudes than others. The lower range of these errors enhances the robustness of our prediction accuracy.

**FIGURE 4:**
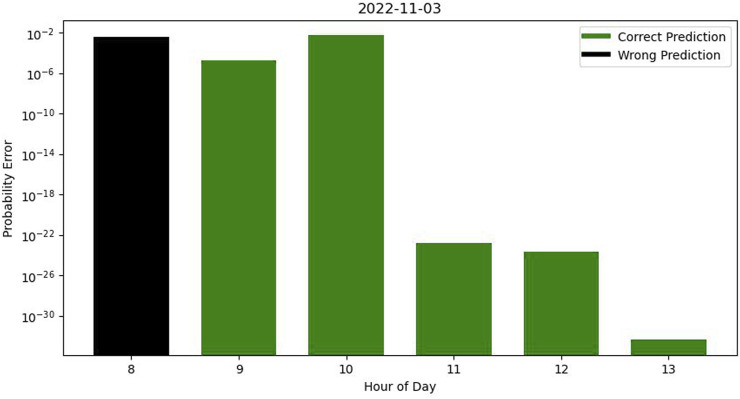
Error graph in a day with some wrong predictions.

Most results in all hours of each separate day are accurate but some wrong predictions occur in our testing dataset. In Figure 4, while most hours were accurately predicted the algorithm failed to correctly predict the occupancy profile during the 8 a.m.–9 a.m. time window.

Furthermore, as described in the experimental data section, different values in absolute differences were considered to identify the most effective approach. After exploring various combinations, it was determined that utilizing a 2-h window between 8:00 and 10:00 on November 1st and 2nd to create the occupied Markov Chain Model was the most successful in predicting occupancy profiles, with an accuracy rate of 97,22%.

### 3.2 Sub-optimal rule results

#### 3.2.1 Real data

The “equal” state was determined to provide the most favorable conditions for producing optimal outcomes using the principles described in Definition 3. By applying various absolute differences between consecutive points for generating the Markov Model’s steady-state matrices, the most effective 2-h training period was identified as 8–10a.m. on 1st November, producing a 13.84 threshold and a 97.22% accuracy rate which was also attained using the first algorithm.

In order to achieve optimal results, it is desirable to minimize the difference between the steady-state probability of the training data and that of the selected testing data set. However, it has been observed that this difference tends to be much higher when the 2-h testing window differs from the training set’s time window, which is 8:00a.m. to 10:00a.m. For instance, in the case where the probability of the occupied state in the training set is 96.73%, and the probability of the occupied state in one of the testing sets within the 8:00 a.m. to 10:00 a.m. time window is 97.24%, the resulting difference is 1.77%. Conversely, when a different 2-h testing window, such as 12:00p.m. to 2:00p.m., is chosen for the same day, the probability of the occupied state is 88.4%, resulting in a difference of 10.6% compared to the training set. These experimental results suggest that the difference could be reduced if each testing set was evaluated using an algorithm trained on the same 2-h window, rather than relying on a fixed training set for all occasions.

As a result, a distinct approach for dividing training sets was utilized in this algorithm. Instead of adopting a 2-h window for training, a specific hour was employed for both training and testing sets. To elaborate, the optimal day (1 November) was selected to train the model from 8:00–9:00a.m., followed by testing against the 8:00–9:00a.m. timeframe for all other days of the month. This procedure was repeated for every working hour, resulting in an accuracy rate of approximately 100%, with certain hours reaching 98% accuracy.

As an illustration, consider dividing the testing data into 30 subsets of 1-h time windows between 12:00 p.m. and 1:00 p.m. In this case, our algorithm will be trained using data from the 12:00 p.m.–1:00 p.m. time window on November 1st. This approach yields perfect classification accuracy, with all 30 subsets correctly identified, and an average difference of 4.44% between the steady-state probabilities of the training and testing sets.

Alternatively, we may choose to use testing data from a fixed time window of 8:00 a.m. to 9:00 a.m. for the entire month. In this scenario, the algorithm trained using data from the same time window of the training set correctly identifies occupancy profiles for 29 out of 30 subsets, with an average difference of 1.8% between the steady-state probabilities of the training and testing sets.

**FIGURE 5:**
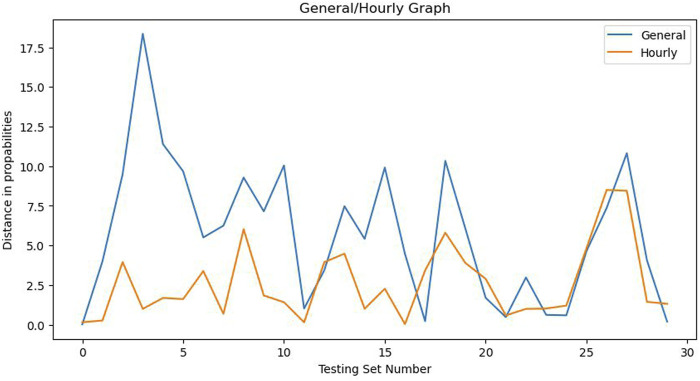
General and Hourly Methods comparison.

When identifying occupancy profiles on a testing set using a different time window, accuracy may be correctly assessed using the general method but the difference between steady state probabilities can vary between 10% and 20% as shown in Figure 5. The hourly approach reduces these errors by a substantial margin.

#### 3.2.2 Experimental data

Notwithstanding the potential efficacy of the technique of splitting hourly data, it cannot be appropriately implemented on the GitHub dataset due to the limitation in the granularity of time sampling, which was at 1-min intervals. The resulting dataset has only 60 data points which are insufficient to adequately evaluate occupancy patterns. Additionally, the dataset captures the presence of only one individual in the room at a time and the absence of this individual can cause further inaccuracies in estimating true occupancy profiles within the limited 60 data points.

A similar splitting method to the procedure of the first algorithm was adopted. We utilized the occupied Markov Model trained on a normal Monday of 16th February 2015 from 09:00 to 16:00, and the unoccupied Markov Model trained on Sunday of 15th February 07:30–16:00. For testing, each of the 15 remaining days was considered, with the time range of 09:00 to 16:00 for each day.

We found that the steady-state algorithm yielded 100% accuracy on all 15 testing sets when 0.8 was set as the threshold for the equal state representing the best difference between two consecutive CO_2_ values. The average difference between the steady-state probabilities of the training and testing sets was 3.4%.

## 4 Discussion

The first and second algorithms demonstrated significant potential in accurately predicting occupancy profiles in both experimental and real case scenarios. Both algorithms easily predicted everything in the experimental data so no further discussion should be made as the experimental data was mostly used to create the algorithms and assess their efficacy. As the real data in the bank of Athens is concerned, both algorithms achieved a 97% accuracy rate. The conditions under which both algorithms were trained were very similar, suggesting that the choice of algorithm can be adapted based on the specifics of the dataset being analyzed, such as sampling data.

An intriguing observation is that out of the five errors that occurred in the real data, some of them took place during similar hours. For instance, three errors occurred on the same day at 8:00 a.m. where the absence of people was incorrectly predicted. Additionally, one false prediction took place on November 19th at 11 a.m. in both algorithms. A possible hypothesis is that the algorithms accurately assessed the unoccupancy of the space at the time, despite it being a working day. One assumption is that the employees did not arrive at that particular time.

For the optimal algorithm, the probability of a faulty classification is computed according to the methodology delineated in Definition 2. The preponderance of accurate predictions is associated with a negligible risk of misclassifying the occupancy profile, given that such an outcome typically registers below the 0.00001% threshold. However, out of the five incorrect predictions the average probability of misclassification equates to approximately 30% which indicates that there was a high chance that the algorithm may have done a mistake which may had been the case.

In the case of the sub-optimal algorithm, the probability of misclassification is deduced by measuring the distance between the probabilities of the steady state as elucidated upon in the sub-optimal rule. By leveraging the Hourly approach, almost all correct predictions manifest only a negligible disparity between the steady-state probability of the testing set and that of the Markov Model.

It is concluded that aside from approximately identical results, the above algorithms also provide similar error profiles for the classification process which further compliments their efficacy. The classification algorithms in Markov chains presented in this paper can be very promising for further research in complex and general situations because of the simplicity, the relatively low computational complexity, and the small state-space of the proposed models (i.e., only 3 states to capture the CO_2_ variability).

**FIGURE 6:**
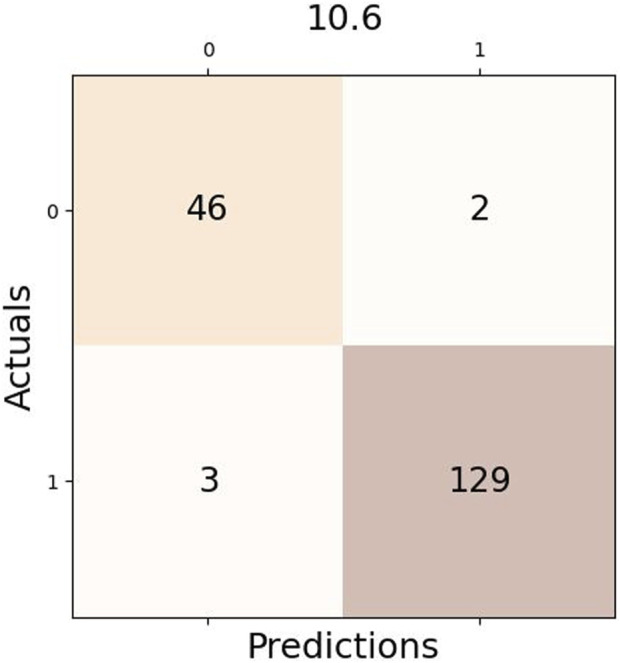
Example of Confusion Matrix using Optimal Rule.

This is the confusion matrix is shown in Figure 6 for one of the two algorithms, which had three true positives (TP) and two false negatives (FN) given a difference of 10.6 between two consecutive CO_2_ values. One of the two false positives is also common among both algorithms, indicating possible commotion on Saturday, even though it was not a working day.

When comparing the General and Hourly methods of creating Markov models, both approaches produced excellent results. The first approach trained the algorithm on 8:00–10:00 a.m. and achieved 97% accuracy even in testing sets with different working hours and presence dynamics. However, the Hourly approach produced slightly better results when the training and testing hours were identical. Specifically, every training and testing hour had 100% accuracy, except for 8:00 a.m. and 2:00 p.m., which had 99% accuracy. This may be due to the fact that people either arrive or leave their workplace and the occupancy profile is not constant throughout the hour. Unfortunately, there is no absolute ground truth available about the total occupancy profile for every hour of each day. The assumptions made in the Introduction section provide a slight solution to this problem since monitoring every single worker or customer in the building was not possible.

The primary emphasis of our current methodology revolves around a data-driven approach which does not encompass factors such as ventilation, varying room characteristics and scalability in more complex scenarios. In our forthcoming work, we aim to develop a more advanced algorithm that incorporates these environmental considerations, providing a comprehensive comparison to the original methodology presented in this paper. We believe that this endeavor will significantly enrich the applicability of our approach.

## 5 Conclusion

In this paper, a model for reproducing presence or absence in a single office or building was used utilizing CO_2_ as the single factor. Both experimental and real data were extracted to create 3 × 3 Markov Chain models and assess the occupancy profile. The algorithms developed in this study demonstrated nearly identical results, exhibiting exceptional accuracy. Further refinement is possible, including the incorporation of parameters not within the scope of this paper such as ventilation and room architecture. The Markov Chain models presented a straightforward yet robust approach for evaluating occupancy profiles. They can be compared or integrated with other algorithms in research endeavors to formulate an even better comprehensive presence detection algorithm. In terms of real-time application, this method proves exceptionally valuable for promptly identifying abrupt shifts in occupancy profiles due to its rapid response time and high-frequency sampling rate. While this paper focused on employing 1-h window testing sets, there exists significant potential for accurately assessing occupancy profiles within shorter minute intervals. In future work, we plan to delve into real-time scenarios leveraging the strengths of this method.

## Data Availability

The raw data supporting the conclusion of this article will be made available by the authors, without undue reservation.

## Footnotes

1 Different results were obtained based on the range used for concluding that the CO_2_ values were in an “equal” state.

2 The careful reader can notice that the next state is completely determined by the current event and the current state.

3
*P*_1_(*P*_2_) are the *a priori* probabilities for HMMs *S* ^1^(*S* ^2^). We assume in our case that *P*_1_ = *P*_2_ = 0.5.

4 Let the steady-state probabilities for HMM *S* ^1^ (*S* ^2^) be denoted by the |*Q*|-dimensional vector $\pi_{ss}^{\left(1\right)}={\left[\pi_{ss,1}^{\left(1\right)},\pi_{ss,2}^{\left(1\right)},\pi_{ss,3}^{\left(1\right)}\right]}^{T}$ (respectively by $\pi_{ss}^{\left(2\right)}={\left[\pi_{ss,1}^{\left(2\right)},\pi_{ss,2}^{\left(2\right)},\pi_{ss,3}^{\left(2\right)}\right]}^{T}$ ). Then, we have $d_{V}\left(\pi_{ss}^{\left(1\right)},\pi_{ss}^{\left(2\right)}\right)=\frac{1}{2}\overset{\left|Q\right|}{\underset{j=1}{\sum }}|\pi_{ss,j}^{\left(1\right)}-\pi_{ss,j}^{\left(2\right)}|$ .
